# Difference in early all-cause mortality among patients having hip arthroplasty a Swedish perioperative registry study 2013–2022

**DOI:** 10.1186/s13018-024-04752-6

**Published:** 2024-05-15

**Authors:** J. Magnusson, J. Karlsson, O. Sköldenberg, J. Albert, C. Frostell, J. G. Jakobsson

**Affiliations:** 1 Karolinska Institutet, Stockholm, Sweden; 2 Sahlgrenska Academy, Institute of Clinical Sciences, Gothenburg University, Gothenburg, Sweden; 3 Department of Orthopaedics, Sahlgrenska University Hospital, Gothenburg, Sweden; 4 Unit of Orthopaedics, Department of clinical sciences at Danderyd Hospital, Karolinska Institutet, Stockholm, Sweden; 5 Department of clinical sciences at Danderyd Hospital, Unit of Anaesthesia and Intensive Care, Karolinska Institutet, Stockholm, SE-182 88 Sweden

**Keywords:** Hip arthroplasty, Cemented, Cementless, Early mortality, Fracture, ASA-class, Anaesthesia

## Abstract

**Introduction:**

Hip arthroplasty is a common orthopaedic procedure worldwide. There is an ongoing debate related to the fixation and anaesthesia impact on the 30-day mortality, particularly in the aging population with higher American Society of Anaesthesiology (ASA) Physical-Status.

**Aim:**

To study the 30-day all-cause mortality in patients undergoing primary hip arthroplasty, with regards to the impact of age, ASA-class, anaesthesia techniques, indication for surgery and fixation techniques.

**Materials and methods:**

Perioperative data for primary hip arthroplasty procedures for osteoarthritis and hip fractures registered in the Swedish Perioperative Registry (SPOR) between 2013 and June 2022 were collected. Binary logistic regressions were performed to assess the impact of age, ASA-class, anaesthetic technique, indication for surgery and fixation on odds ratio for 30-day mortality in Sweden.

**Results:**

In total, 79,114 patients, 49,565 with osteoarthritis and 29,549 with hip fractures were included in the main study cohort. Mortality was significantly higher among hip fracture patients compared with osteoarthritis, cumulative 8.2% versus 0.1% at 30-days respectively (*p* < 0.001). Age above 80 years (OR3.7), ASA 3–5 (OR3.3) and surgery for hip fracture (OR 21.5) were associated with significantly higher odds ratio, while hybrid fixation was associated with a significantly lower odds ratio (OR0.4) of 30-day mortality. In the same model, for the subgroups of osteoarthritis and hip fracture, only age (OR 3.7) and ASA-class (OR 3.3) had significant impact, increasing the odds ratio for 30-day mortality. Hemi arthroplasty was commonly used among the hip fracture patients 20.453 (69.2%), and associated with a significantly higher odds ratio for all-cause 30-day mortality as compared to total hip arthroplasty when adjusting for age and ASA-class and fixation 2.3 (95%CI 1.9–2.3, *p* < 0.001).

**Conclusions:**

All-cause 30-day mortality associated with arthroplasty differed significantly between the two cohorts, hip fracture, and osteoarthritis (8.2% and 0.1% respectively) and mortality expectedly increased with age and higher ASA-class. Anaesthetic method and cement-fixation did not impact the odds ratio for all-cause 30-day mortality after adjustment for age and ASA-class.

## Introduction

Hip arthroplasty (or hip replacement surgery) is one of the most frequent and effective orthopaedic procedures worldwide. More than 1 million procedures are performed each year [1], and in the majority of osteoarthritis patients with a great improvement in the quality of life [2]. The impact on quality of life for the hip fracture patient is not as well studied. The majority of hip arthroplasties are performed on the indication of osteoarthritis, followed by fracture of the femoral neck, while other reasons such as vascular necrosis, dysplasia, and inflammatory arthritis are less frequent [1, 3]. Given an aging population with potentially more comorbidities, a significant rise in the number of procedures is anticipated [4]. . The need for hip arthroplasty will most certainly increase both for patients with osteoarthritis and hip fractures. Nemes et al.‘s analysis, utilizing data from the Swedish Hip Arthroplasty Register, predicts a substantial increase in the need for such procedures in Sweden [5].

There are several aspects that needs to be considered when the perioperative quality of care associated with hip arthroplasty is assessed. Early 30-day mortality is a commonly used and referred quality indicator [6–8]. There are several studies that have been published in recent years assessing early mortality associated with hip arthroplasty for osteoarthritis. Taken as a whole there is no clear clinical difference between cemented and cementless hip arthroplasties in terms of mortality [9–12]. There are also several recent studies that have assessed hip arthroplasty for the treatment of hip fractures. The crude mortality has in several of these studies showed a higher early mortality rate associated with cemented hip arthroplasty [13–17]. Studies have mostly focused on elderly patients. These studies have shown no early mortality difference for osteoarthritis hip arthroplasty but a high crude risk for early mortality for hip fracture hip arthroplasty [18, 19]. The anaesthetic technique, either general or neuraxial is also a matter of discussion. Three recent meta-analyses, have consistently found no significant differences in mortality outcomes when comparing regional and general anesthesia [20–22]. While Memtsoudis et al. examined a broad spectrum of orthopaedic conditions such as osteoarthritis, rheumatoid arthritis, trauma, and infectious diseases, Kunutsor et al. and Chen et al. directed their analysis towards patients undergoing hip fracture surgery, none of these found significant difference in mortality between neuraxial and general anaesthesia.

These findings underscore the importance of evaluating anaesthesia techniques and use of cement fixation within the context of specific surgical indications and patient populations. There is no recently published data related to the all cause 30-day mortality associated with hip arthroplasty. Studies showing patient profiles, anaesthesia, fixation practice, with a focus on the cemented technique and 30-day mortality associated with hip arthroplasty for the osteoarthritis as well as the hip fracture are missing.

The current study aimed to evaluate the 30-day mortality rates following hip arthroplasty surgery for either osteoarthritis or hip fracture.

## Materials and methods

This study is a nation-wide, registry-based, retrospective cohort study, where data from the Swedish Perioperative Registry (SPOR) were collected.

SPOR, established in 2011, is one of many quality registers in Sweden [23]. SPOR is supported by the National Board of Health and Welfare, Swedish Association for Anaesthesia & Intensive care^1^. It has a high coverage rate during recent years, and it now contains a total of 159 variables related to the perioperative care, starting with planning for surgery until discharge from the recovery room and additional automatic control of death by interlinking data files with the national death register. The register was recently validated and found to be accurate in overall terms [23].

### Patients

The data consists of information on all patients who underwent either cemented, hybrid or cementless hip arthroplasty in Sweden between 2013 and June 31st, 2022. The study population was selected based on specific inclusion criteria; patients who were 18 years or older, and had complete data related to age and ASA-class and had undergone a hip arthroplasty procedure with the following International Classification of Diseases, Tenth Revision (ICD-10) procedure codes (corresponding Nordic Medico-Statistical Committee (NOMESCO) Classification of Surgical Procedures codes [24] included) : NFB09 (primary half or partial hip joint arthroplasty without cement, NOMESCO NFB0y), NFB19 (primary half or partial hip joint arthroplasty with cement, NOMESCO NFB1y ), NFB29 (primary total hip arthroplasty without cement, NOMESCO NFB20), NFB39 (primary total hip arthroplasty with hybrid technology i.e., with one component cemented and one without, NOMESCO NFB30), and NFB49 (primary total hip arthroplasty with cement, NOMESCO NFB40). Exclusion criteria for the study were patients with missing information about age, ASA-class, procedural codes, and errors in time (days) to death. Patients were only included once, if they have undergone repeated procedures only the last was included in the mortality analysis.

### Primary objective

30-day all-cause mortality.

#### Exposure

Difference between patients having arthroplasty because of osteoarthritis and hip fracture adjusted for age, ASA-class, and anaesthetic technique.

#### Confounding/bias

Age, ASA-class, anaesthetic technique and fixation technique, focus on the use of cement.

### Secondary objective

Comparison of 30-day all-cause mortality between cemented, hybrid and cementless hip arthroplasty, for osteoarthritis and fracture; indicated hip arthroplasty adjusted by age, ASA-class, and anaesthetic techniques.

### Variables

The analysed variables included 30-day mortality, hip arthroplasty fixation (cemented, cementless, hybrid), age, ASA-class, sex, cause of surgery, anaesthesia technique, duration of surgery, duration of anaesthesia, and duration of recovery room stay.

The indication for surgery was classified based on ICD-10 codes for osteoarthritis (M160-169) and fracture (S720, S7200, 7201, 7210, 722, 7220, 723, 7230, 7231, 724, 7270, 728, 7280, 729, 7290). Fixation techniques, namely cemented, hybrid, and cementless hip arthroplasty, were classified by grouping the ICD-10 codes for procedures using cemented fixation technique (NFB19, NFB49) in one group, hybrid technique (NFB39) in another, and procedures using cementless fixation technique (NFB09, NFB29) in a third group. Hemi arthroplasty was defined by NFB09 and NFB19. Age was categorized as 18–65, 66–80, and > 80 years. Sex was classified as male or female. ASA-class was divided into classes 1–2 and classes 3–5. Anaesthesia techniques were categorized as general anaesthesia, neuraxial anaesthesia, and combination (general + neuraxial), with other low-prevalence anaesthesia techniques (488 cases, 11 different anaesthesia techniques or combinations) classified as missing. Mortality was classified as binary (alive or deceased) for each time frame.

### Statistical analysis

Numerical data, such as age and time events, are presented as mean and standard deviation (SD), while categorical data, such as sex, age groups, ASA-class, and perioperative findings, are presented as numbers and percentages (%). The distribution of continuous numerical variables was analysed by reviewing the data collected as a histogram. Comparison between groups was performed with independent t-test for numerical continuous variables and Chi-2-test for categorical data. 95% confidence interval was also calculated for the proportion of all-cause 30-day mortality associated to the procedures studied by the costume table output in the SPSS software. A binary logistic regression was performed to assess the Odds Ratio and 95% confidence interval for the Odds Ratio for all-cause 30-day mortality associated with cemented or cementless arthroplasty unadjusted and adjusted for sex, age and ASA-class, and anaesthesia technique. A binary logistic regression was also performed to assess the Odd Ratio for all-cause 30-day mortality associated to hemi arthroplasty in the hip fracture patient adjusted for age and ASA-class, anaesthetic technique and fixation. A p-value < 0.05 was considered statistically significant. All data were managed in Microsoft 365 Excel, and the statistical analysis was conducted using SPSS 28 software.

**Fig. 1:**
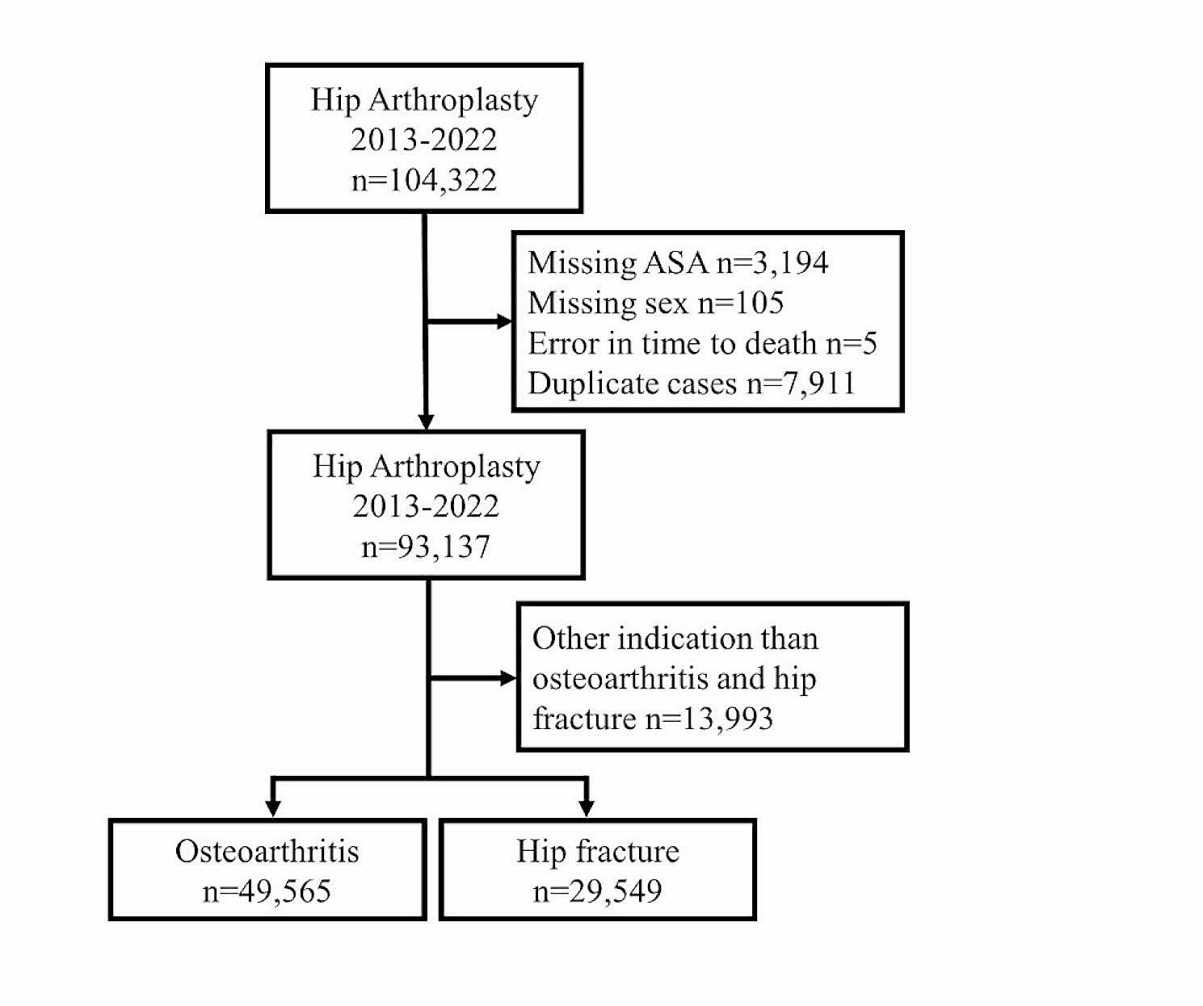
Patient flow-chart

## Results

In all 79,114 patients were included in the main study cohort (Fig. 1). Table 1 presents patient characteristics, anaesthetic technique and all-cause 30-day mortality for the 6 subgroups analysed. The mean age for the entire cohort was 74 SD 11.4, a majority 47,508 (60%) females and 31,606 (40%) male, ASA-class 2 was the most frequent seen 40,371 (51%). Females were older than males 75 SD 11 versus 73 SD 11 respectively (*p* < 0.001). The proportion of ASA 3–5 for the entire cohort was 3% higher than ASA 1–2 (*p* < 0.001).

Comparing osteoarthritis and hip fractures showed the hip fracture patients were significantly older. There were more female patients’ 19,066 out of 29,549 (64%) compared with 28,442 out of 49,565 (57%), (*p* < 0.001) and had higher proportion ASA-class 3–5, 18,436 out of 29,549 (62%) versus 11,055 out of 49,565 (22%), (*p* < 0.001).

Neuraxial anaesthesia with general anaesthesia/sedation or only neuraxial anaesthesia was overall the predominant anaesthetic technique 37,129 (82%) compared with only general anaesthesia 8,360 (18%), (*p* < 0.0001). Among the osteoarthritis patient general anaesthesia was significantly less commonly used among patient having cemented arthroplasty.

Cemented fixation was the predominant fixation technique used in 58,711 patients (74%), followed by cementless fixation in 13,083 (17%), and hybrid fixation in 7,321 (9%).

### Mortality

The overall 30-day mortality was 338 (0.4%) within 24 h, 993 (1.3%) during the first week postoperative week and 2,471 (3.1%) within 30 days.

### Subgroup analysis

Mortality was significantly higher in hip fracture patients compared with osteoarthritis at 24-hours, 7-days, and 30-days, 1.1% vs. 0.0% at 24-hours, 3.3% vs. 0.0% at 7-days, and 8.2% vs. 0.1% cumulative at 30 days respectively (*p* < 0.001).

Mortality was also significantly higher for cemented arthroplasty when analysing the entire cohort, cemented all-cause 24-hour mortality was 379 out of 69,215 (0.5%), hybrid 2 out of 8,382 and cementless 3 out of 15.510, 30-day mortality was 2,438 out of 58,711 (4.2%), hybrid, 9 out of 7,321 (0,1%) and cementless, 24 out of 13,082 (0.2%) (*p* < 0.001).

Cemented arthroplasty had the highest 30-day mortality when dividing into the two subgroups: osteoarthritis and hip fracture patients, 0.2% and 8.2% respectively (Table 1).

**Table 1:** Patients demographics, anaesthetic techniques used and early postoperative mortality for the 2 main group of patients studied, hip arthroplasty for osteoarthritis and hip fracture. Number of patients (%) and for the main outcome all-cause mortality number of patients (%, 95%Confidence interval)

	Osteoarthritis *N* = 49,565					Hip fracture *N* = 29,549				
	*Cemented* *N* = 29,670	*Hybrid* *N* = 7,087	*Cementless* *N* = 12,808	**All** *N* = 49,565	*P-value*	*Cemented* *N* = 2,9041	*Hybrid* *N* = 234	*Cementless* *N* = 274	**All** *N* = 29,549	*P-value*
Age years, mean ± SD	74 ± 7	69 ± 10	60 ± 10	70 ± 10		82 ± 9	71 ± 10	75 ± 14	82 ± 9	
**Age-class** no (%)					**< 0.001**					**< 0.001**
18–65 years	3,414 (12)	2,393 (34)	9,014 (70)	14,821 (30)		1,185 (4)	70 (30)	76 (278)	1331 (5)	
65–80 years	20,195 (68)	3,980 (56)	3,657 (29)	27,832 (56)		10,460 (36)	133 (57)	85 (31)	10,678 (36)	
Above 80 years	6,061 (20)	714 (10)	137 (1)	6,912 (14%)		17,396 (560)	31 (13)	113 (41)	17,540 (59)	
**Sex**					**< 0.001**					0.153
Female	18,787 (63)	4,166 (59)	89 (43)	28,442 (57)		18,758 (65)	145 (62)	163 (560)	19,066 (65)	
Male	10,883 (37)	2,921 (41)	7,319 (57)	21,123 (43)		10,283 (35)	89 (38)	111 (40)	10,483 (35)	
**ASA-class** no (%)					**< 0.001**					**< 0.001**
1–2	21,725 (73)	5,615 (79)	11,170 (87)	3,8510 (78)		10,819 (37)	150 (64)	144 (53)	11,113 (38)	
3–5	7,945 (27)	1,472 (21)	1,638 (13)	11,055 (22)		18,222 (63)	84 (36)	130 (47)	18,436 (62)	
**Ane technique** no (%)					**< 0.001**					0.319
Ga	2,889 (17)	1,131 (22)	1,631 (22)	5,633 (19)		2,664 (17)	29 (21)	34 (21)	2,727 (17)	
Neuraxial	4,839 (29)	1,370 (27)	2,055 (27)	8,264 (28)		7,222 (46)	58 (41)	77 (47)	7,357 (46)	
Combination	9,064 (54)	2,604 (51)	3,813 (51)	1,5481 (53)		5,922 (37)	53 (38)	52 (32)	2,027 (37)	
**Deceased within 24 h** no (%)	3 (0.0)	0 (0.0)	0 (0.0)	3 (0.0)	0.366	331 (1.1)	1 (0.4)	3 (1.1)	335 (1.1)	0.590
**Deceased within 7 days** no (%)	19 (0.1)	3 (0.0)	2 (0.0)	24(0.0)	0.111	961 (3.3)	1 (0.4)	7 (2.6)	969(3.3)	**0.038**
**Deceased with** **30 days** no (%; 95%CI*)	49 (0.2; 0.10–0.20)	7 (0.1; 0.00-0.2 )	6 (0.05; 0.02–0.1)	62 (0.1; 0.1–0.2)		2389 (8.2; 7.91–8.55)	2 (0.9; 0.18–2.71)	18 (6.6; 4.08–9.97)	2409 (8.2; 7.8–8.5)	

*Proportions Confidence interval, SD standard deviation, Ane: anaesthetic technique, GA: general Anaesthesia, ASA: American Society of Anesthesiology

### Logistic regression

Tables 2, 3 and 4 show the unadjusted and adjusted odds ratio for all-cause 30-day mortality.

Table 2 shows the overall cohort and the significant impact of age above 80 years and ASA classes 3–5 on early mortality. Anaesthetic technique did not have any significant impact on the adjusted model, where adjustments for age, ASA-class, indication and fixation were made. The odds ratio was highest for patients who had sustained a fracture. Furthermore, cementless group had significantly lower OR in the unadjusted model, that was not seen in the adjusted model.

**Table 2:** Unadjusted and adjusted Odds Ratio for all-cause 30-day mortality among patients having hip arthroplasty for the entire cohort, osteoarthritis, and hip fracture

	Unadjusted			Adjusted		
	Odds ratio	95%CI	*p*-values	Odds ratio	95%CI	*p*-values
**Age**						
18 to 65 years	-			-		
66 to 80 years	5.7	4.1; 8.1	**< 0.001**	1.4	0.9; 2.2	0.12
> 80 years	40.2	28.7; 56.2	**< 0.001**	3.7	2.4; 5.8	**< 0.001**
**ASA-class**						
1 and 2	-			-		
3 to 5	10.0	9.0; 11.2	**0.000**	3.3	2.8; 3.8	**< 0.001**
**Anaesthetic technique**						
GA	-			-		
NA	1.44	1.2; 1.7	**< 0.001**	1.0	0.9; 1.2	0.5
Comb	0.7	0.6; 0.8	**< 0.001**	0.9	0.7; 1.0	0.1
**Indication**						
Osteoarthritis	-			-		
Fracture	70.9	55.1; 91.1	**< 0.001**	21.5	15.2; 30.2	**< 0.001**
**Fixation technique**						
Cemented	-			-		
Hybrid	0.02	0.02; 0.05	**< 0.001**	0.4	0.2; 0.9	**0.035**
Cementless	0.04	0.03; 0.06	**< 0.001**	0.8	0.4; 1.4	0.4

CI confidence interval, ASA American Society for Anesthesiology, GA general anaesthesia, NA neuraxial anaesthesia

Table 3 shows the regression results from the osteoarthritis subgroup and the similar significant impact of age above 80 years and ASA-classes 3–5. Anaesthetic technique and fixation did not have any significant impact neither unadjusted or in the adjusted model, where we adjusted for age, ASA-class, anaesthesia, and fixation.

**Table 3:** Unadjusted and adjusted Odds Ratio for all-cause 30-day mortality among patients having hip arthroplasty for osteoarthritis

	Unadjusted			Adjusted		
	Odds ratio	95%CI	*p*-values	Odds ratio	95%CI	*p*-values
**Age**						
18 to 65 years	-			-		
66 to 80 years	1.6	0.7; 3.6	0.3	0.9	0.2; 3.0	0.8
> 80 years	8.0	3.7; 17.6	**< 0.001**	4.4	1.3; 15.5	**0.017**
**ASA-class**						
1 and 2	-			-		
3 to 5	5.9	3.5; 9.9	**< 0.001**	5.0	2.5; 10.0	**< 0.001**
**Anaesthetic technique**						
GA	-			-		
NA	2.2	0.9; 5.4	0.1	1.7	0.7; 4.3	0.2
Comb	0.8	0.3; 2.2	0.8	0.8	0.3; 2.2	0.7
**Fixation technique**						
Cemented	-			-		
Hybrid	0.6	0.27; 1.3	0.2	2.2	0.6; 8.3	0.3
Cementless	0.3	0.1; 0.7	0.3	0.9	0.2; 4.7	0.9

CI confidence interval, ASA American Society for Anesthesiology, GA general anaesthesia, NA neuraxial anaesthesia

Table 4 shows the regression analysis for the hip fracture subgroup and show similarly significant impact of age above 80 years and ASA-classes 3–5. Anaesthetic technique and fixation did not have any significant impact, neither unadjusted nor in the adjusted model, where adjustments for age, ASA-class, anaesthesia technique, and fixation were made.

**Table 4:** Unadjusted and adjusted Odds Ratio for all-cause 30-day mortality among patients having hip arthroplasty for hip fracture

	Unadjusted	Adjusted				
	Odds ratio	95%CI	*p*-values	Odds ratio	95%CI	*p*-values
**Age**						
18 to 65 years	-			-		
66 to 80 years	2.1	1.4; 3.1	**< 0.001**	1.4	0.8; 2.3	0.2
> 80 years	6.0	4.1; 8.8	**< 0.001**	3.5	2.2; 5.7	**< 0.001**
**ASA-class**						
1 and 2	-			-		
3 to 5	3.8	3.4; 4.3	**< 0.001**	3.2	2.8; 3.7	**< 0.001**
**Anaesthetic technique**						
GA	-			-		
NA	1.0	0.8; 1.1	0.8	1.0	0.9; 1.2	0.7
Comb	0.8	0.6; 0.9	**0.002**	0.9	0.7; 1.0	0.1
**Fixation technique**						
Cemented	-			-		
Hybrid	0.1	0.02; 0.4	**< 0.001**	0.2	0.1; 1.5	0.9
Cementless	0.8	0.5; 1.3	0.3	1.0	0.5; 1.8	0.2

CI confidence interval, ASA American Society for Anesthesiology, GA general anaesthesia, NA neuraxial anaesthesia

#### Hemi versus total and hybrid arthroplasty

Hemi hip arthroplasty was used in overall 23,204 of all patients studied (24.9%). There was a huge difference in it use, hemi arthroplasty was used in 20.453 (69.2%) out of the 29.549 fracture patients and in 45 (0.1%) of osteoarthritis patients (*p* < 0.001).

All 45 osteoarthritis patients having a hemi arthroplasty were alive day 30. Hemi arthroplasty was among the hip fracture patients associated with a significantly higher all-cause 30-day mortality as compared to total and hybrid arthroplasty, 2,180 deceased out of the 20,453 (10.7%) hemi arthroplasty patients as compared to 229 out of 9, 096 total and hybrid patients (2.5%) (*p* < 0.001). Hemi arthroplasty was among the hip fracture patients, associated with a significantly higher odds ratio when adjusting for age and ASA-class, anaesthetic technique, and fixation technique see Table 5.

**Table 5:** Adjusted Odds Ratio for all-cause 30-day mortality among patients having hip arthroplasty for hip fracture, adjusted for age and ASA class, anaesthetic technique, fixation and type of arthroplasty, total vs. hemi

	Adjusted		
	Odds ratio	95%CI	*p*-values
**Age**			
18 to 65 years	-		
66 to 80 years	1.4	0.8; 2.3	0.2
> 80 years	3.5	2.2; 5.7	**< 0.001**
**ASA-class**			
1 and 2	-		
3 to 5	3.2	2.8; 3.7	**< 0.001**
**Anaesthetic technique**			
GA	-		
NA	1.0	0.9; 1.2	0.7
Comb	0.9	0.7; 1.0	0.1
**Fixation technique**			
Cemented	-		
Hybrid	0.2	0.1; 1.5	0.9
Cementless	1.0	0.5; 1.8	0.2
**Prosthesis**			
Total hip arthroplasty	-		
Hemi hip arthroplasty	2.3	1.9; 2.8	**< 0.001**

CI confidence interval, ASA American Society for Anesthesiology, GA general anaesthesia, NA neuraxial anaesthesia

## Discussion

This quality register-based (SPOR) based study had a primary aim to assess all-cause 30-day mortality associated with hip arthroplasty procedures during recent years in Sweden, taking age, ASA-class, indication for the surgical procedure, anaesthetic technique, and fixation, the use of cement, into account. Early mortality was as expected significantly higher in the hip fracture cohort compared with the osteoarthritis group, 8.2 vs. 0.1% respectively. In this context, it important to remember that these two sub-cohorts are different in terms of general health and co-morbidity. Anaesthetic techniques as well as cemented fixation were after adjustment not associated with increased odds ratio for early mortality. Age above 80 years and ASA classes 3–5 had, however, significantly higher odds ratio for all cause 30-day mortality in the total cohort as well as in the two subgroups, osteoarthritis, and hip fractures.

### Differences between indications

As anticipated, a significant disparity in mortality rates was observed between the two primary cohorts: those with osteoarthritis and those with fractures. This difference was largely attributed to variations in demographics, specifically age and ASA-class. The mean age difference of twelve-years and a 40% discrepancy in the proportion of patients classified as ASA 3–5 is most certainly of major importance. Unfortunately, the SPOR register does not encompass fragility scores, which could provide further insights into these differences,

The hip fracture group was associated with a 21.5 higher odds ratio for all cause 30-day mortality as compared with the osteoarthritis group in the adjusted model. The mortality among the fracture patients is similar to what was found in an earlier study, assessing all hip fracture surgery 2016 and 2017 [25]. The mortality following hip arthroplasty in the present study is also in line with a study by Maceroli et al. from US [26]. They found a 30-day mortality after hemiarthroplasty of 8.4% compared with 5.7% after total hip arthroplasty in hip fracture patients above 60 years of age. The present results in terms of the hip fracture mortality are also similar to the results in a Norwegian study that assessed the impact of a fast-tracking concept in hip fracture patients [27]. These researchers found a 7.9% versus 6.5% 30-day mortality for the conventional and fast-tracked groups respectively. It should be acknowledged that only approximately 50% of the patients underwent an arthroplasty procedure, either hemi- or total arthroplasty.

A study by Pedersen at al. based on the Nordic arthroplasty register assessing mortality following total hip arthroplasty for primary osteoarthritis found a 30-day mortality in the 188,606 patients included to be 0.21% for cemented total hip arthroplasty and 0.12%) for cementless fixation. This is similar to the osteoarthritis cohort in the present study (0.12%) [12], . Large differences in the one-year mortality between patients having undergone arthroplasty for either osteoarthritis or hip fracture were also observed in a recent German Arthroplasty-based study by Szymsky et al. [17], 12.6% compared with 3% respectively.

There are several potential factors contributing to the large difference in early mortality. Age and ASA-class are of course of importance, but fragility is most certainly an important factor in combination with the trauma as such and co-morbidity. It is also important to recognize that the mortality rates associated with osteoarthritis may be somewhat overstated. This potential discrepancy could stem from the fact that some private units, which likely treat a healthier cohort of patients, may not fully report their data to the SPOR register, particularly in the earlier time period of this cohort. Consequently, the observed difference in mortality rates between patients with fracture indications and those with osteoarthritis could, in reality, could be even more pronounced.

We found that a majority of arthroplasties performed in fracture patients were hemi arthroplasty. The hemi arthroplasty was among these fracture patients associated with a higher all-cause mortality, and the odds ratio for all-cause 30-day mortality was still significantly higher as compared to total and hybrid arthroplasty after adjusting for age and ASA-class, anaesthetic and fixation technique (OD 2.2). This is a finding in line with the study by done on the Swedish hip register published in 2018 [28].

### Differences between fixation techniques

The study by Pedersen et al. [12] study found no significant differences in mortality between cemented and cementless arthroplasty in patients with osteoarthritis. This is in harmony with the present study. A study by Ekman et al. [10] from Finland including 62,221 hip arthroplasties for osteoarthritis found in a similar manner no differences in early and postoperative mortality between cemented and cementless fixations. Lindberg-Larsen et al. [18] studied patients with osteoarthritis above 70 years of age based on data from the Danish hip arthroplasty register. They found similar risks of mortality within 30 days (0.2% vs. 0.3%) in these elderly patients. Ricksten et al. [14] studied patients having undergone hip arthroplasty due to femoral neck fractures and found a significantly different 30-day mortality, with 3% in the cementless group and 9% in the cemented group (*p* = 0.03). This is also in harmony with the results of the present study. Tsai et al. [19] studied patients aged 80 and above who underwent cemented hip arthroplasty and they reported a higher 30-day mortality. Interestingly, Kristensen et al. [13] in a Norwegian register-based study found no mortality differences at one-year between cemented and cementless arthroplasties after hip fractures. Fernandez et al. [15] presented results from a prospective randomized trial that compared cemented and cementless hemiarthroplasties in 1,215 patients 60 years and above with hip fracture. They found modest, but significantly better quality of life and lower risk for periprosthetic fractures in the cemented group. The one-year mortality was 23.9% in the cemented group and 27.8% in the uncemented group. Springer et al. [16] and Kheir et al. [29] found likewise in register-based studies from US a clear reduction in the risk of revision in patients having undergone cemented arthroplasty for hip fracture patients. Springer et al. also found that stem cemented fixation was associated with an increased odds ratio of in-hospital, 90-day and one-year mortality.

The most similar study to the present one was published by Dale et al. [11] covering the time period 2005 to 2018, using data from the Norwegian Arthroplasty Register. It included in a similar manner both osteoarthritis and hip fracture patients, and different fixation techniques. They found that all modes of fixation had similar adjusted 3-day, 30-day, 90-day mortality risks. The only difference compared with the present study were the lower odds ratio for hybrid fixation, as shown by the regression analysis. These results should, however, be put into perspective, considering that the hybrid fixation technique in the present study was used in only a limited proportion of patients, i.e., only 9% in the entire cohort.

### Differences in anaesthetic techniques

There were no differences in mortality associated with anaesthetic techniques after adjustment for age, ASA, indication and fixation technique. This finding is in line with a previous study assessing mortality after hip fracture surgery based on SPOR data [25]. The International Consensus on Anaesthesia-Related Outcomes after Surgery group (ICAROS) published a systematic review and meta-analysis in 2019 [30]. The consensus statement, based on the meta-analysis, was a recommendation for neuraxial anaesthesia for hip arthroplasty, but did also comment that the evidence level was moderate to low. The REGAIN study published 2021 including patients 50 years of age or older who underwent surgery for hip fractures at 46 U.S. and Canadian hospitals, did not find any differences between general and spinal anaesthesia, with respect to survival and recovery of ambulation at 60 days [31]. A more recent systematic review studied the anaesthesia impact on multiple outcomes after hip fracture surgery. This study did now show any differences between spinal and general anaesthesia, except for patients with renal impartment. Mortality did not differ either [21].

### Strength and limitations

The present study is based on the Swedish perioperative register SPOR. It covers the period 2013 until the first half year of 2022. It includes all primary hip arthroplasties operated on due to osteoarthritis or hip fractures, age 18 years or more during the period covered. It covers patients operated on in university hospitals as well as regional and standard hospitals, thus both in high volume centres and low volume centres. The mortality is based on the collective consignment between the SPOR and Swedish death register and should thus present robust mortality data. One should also keep in mind that Sweden has a tax funded health care system and patients of all ages, and with comorbidities and sociodemographic background undergo surgery on indication-based premises.

There are also limitations to this study. There was a surprisingly high number of missing data related to the anaesthetic technique. We did not include the time to surgery for the hip fracture group of patients or to what extent enhanced recovery after surgery protocols have been implemented.

The present study shows a large difference in early mortality between the two main subgroups, osteoarthritis, and fracture patients. We had an interest in assessing current early mortality and how much of a difference that could be seen between the two major groups of hip arthroplasty in Sweden and the impact of cement use. The early mortality was expectedly associated with higher age and ASA-class, while anaesthetic technique and fixation appear to have minor impact. The more than 8% 30-day mortality among the hip fracture patients, although in line with mortality seen among hip fracture patients, calls for a deeper analysis. It should also be acknowledged that the fracture group of patients include 50 patients with ICD-10 codes (723, 7230, 7231, 724, 7270, 728, 7280, 729, and 7290) covering any types of femur fractures, but all of these patients had a hip arthroplasty. Whether these patients had undergone also more distal procedures cannot be assessed as further procedural codes were not available in the data set.

The register does not contain information about medical history and current medications and information about the cause of death is not available. The register does not contain information related to complications during the hospital stay after discharge from the recovery room and information about return to hospital needs. Assessment of fragility and activities of daily living is also lacking. The combination of advanced age, a high ASA-class, and fragility undeniably plays a critical role in determining mortality outcomes following intermediate surgical procedures like hip arthroplasty. This triad significantly impacts patient prognosis and highlights the need for meticulous preoperative assessment and optimisation. Therefore, it is of great importance to further analyse patient factors and if possible, improve the perioperative care. The choice of hemi vs. total arthroplasty for the hip fracture patients’ needs also further studies. The increased early mortality seen in the present study must be put in context of the benefits associated with in use in a longer context [32].

## Conclusion

All-cause 30-day mortality associated with arthroplasty procedure differed significantly between the two cohorts, hip fractures, and osteoarthritis (8.2% and 0.1% respectively). Anaesthetic technique and cemented fixation did not impact the odds ratio for all-cause 30-day mortality after adjustment for age and ASA-class. Current Swedish practice related to the anaesthesia and fixation for hip arthroplasty appears to be adequate in terms of early mortality for both osteoarthritis and hip fracture procedures. Efforts to improve the overall care of the hip fracture patients is, however, of major importance in future years.

## Data Availability

Data is available on request according to National regulations.

## Footnotes

1 https://spor.se/.
